# Supplementary material for: A Comparison between Enrichment Optimization Algorithm (EOA)-Based and Docking-Based Virtual Screening
Source: Int J Mol Sci. 2021 Dec 21;23(1):43. doi: 10.3390/ijms23010043 (PMC8744642; doi:10.3390/ijms23010043)
Supplement: Supplementary file 1 [file ijms-23-00043-s001.zip › ijms-1501928-supplementary.pdf]

Supplementary Information

A comparison between Enrichment Optimization Algorithm (EOA)-based and docking-based virtual screening

Authors: Jacob Spiegel and Hanoch Senderowitz*

Affiliation: Department of Chemistry, Bar-Ilan University, Ramat-Gan 5290002, Israel

* Corresponding author: Hanoch Senderowitz, Email: hsenderowitz@gmail.com

Description of the modified EOA algorithm:

Given a dataset of *M* compounds (of which *L* are active and *O* are inactive), characterized by *N* descriptors:

1. Select $\{X_i\}_{i=1,k}$ random descriptors. Note that *k* is a subset of *N*.
2. Select $\{C_i\}_{i=1,k}$ random weights.
3. For each compound calculate a predictive activity value: $A_j = \sum_{i=1}^{k} X_i C_i$.
4. Sort $\{A_j\}_{j=1,M}$, from highest to lowest.
5. Calculate the primary score by counting the number of known actives, within the first *L* places of the sorted list. Call this score P_1.
6. Independently normalize the indices of the *L* active compounds and the *O* inactive compounds. Designate the normalized indices as *j'* and *j''*, respectively.
7. Sum the normalized *j'* indices for the active compounds with ranks > *L*, $S_{active} = \sum_{v=1}^{O} j'_v$.
8. Sum the normalized *j''* indices for the inactive compounds with ranks < *L*, $S_{inactive} = \sum_{u=1}^{L} j''_u$.
9. Calculate the secondary score as $S_1 = S_{active} - S_{inactive}$
10. Normalize S_1 to the range [0-1] by means of an inverse sigmoid function of the form: $(S_1) = \frac{1}{1+e^{C \times S_1}}$, where $C = 0.07$.
11. Calculate the total score as $T_1 = P_1 + \mathit{normalized}\ S_1$.
12. Optionally select new descriptors with new weights and/or modify the weights of the current descriptors so that $C_i^{new} = C_i^{old} \pm \Delta J$, where, $\Delta J = \text{random number between specific ranges.}$
13. Calculate $A_j^{new} = \sum_{i=1}^{k} X_i C_i^{new}$ or $A_j^{new} = \sum_{i=1}^{k} X_i^{new} C_i^{new}$
14. Sort $\{A_j^{new}\}_{j=1,M}$, from highest to lowest.
15. Calculate T_2 according to steps (5-11).
16. If $T_2 > T_1$, accept and set: $T_1 = T_2$; $X_i^{old} = X_i^{new}$; $C_i^{old} = C_i^{new}$.
17. If $T_2 \leq T_1$, accept according to the Metropolis MC criterion:
18. A number, *r*[0,1] is generated randomly and the step is accepted if $r < e^{-\frac{\Delta E}{RT}}$, where $\Delta E = T_1 - T_2$. When using MMC simulations to obtain the canonical ensemble, *R* is the gas constant and *T* is the absolute temperature. When using MMC as global optimizer as in the present case, *R* and *T* are constants with no physical meaning and their values simply determine the acceptance rate. In the present case, the term *RT* was linearly reduced in accord with the simulated annealing procedure.
19. If the step is rejected, keep the old values of the descriptors and weights.
20. Go back to step 12.

Table S1. 1D and 2D descriptors calculated by the Canvas program.

Descriptor Family	1D/2D	# descriptors
Physicochemical	1D & 2D	365
Topological	2D	219
LigFilter	1D & 2D	170

Table S2. A list of the most common descriptors appearing in all EOA equations derived for each dataset. For each descriptor, the number of occurrences and a short description is provided.

Dataset	Descriptor	#occurences	Comment
ACES	MR1	9	Molar refractivity
	Balaban-type index from Z weighted distance matrix - Barysz matrix	8	Balaban Index
	ssCH2_Avg	6	Average value of the ssCH2 electrotopological state index
	Ring bridge count	6	Number of ring bridges in the molecule
HIVPR	Neutral acceptor groups	7	Number of neutral acceptor groups in the molecule
	sOm_Key	7	Presence of the sOM (electrotopological state index nomenclature) moiety in the molecule
	sssN_Key	7	Presence of the sssN (electrotopological state index nomenclature) moiety in the molecule
	aaCH_Avg	6	Average value of the aaCH electrotopological state index
	aaCH_Cnt	6	Number of the aaCH (electrotopological state index nomenclature) moieties in the molecule
	dssC_Cnt	6	Number of the dssC (electrotopological state index nomenclature) moieties in the molecule
	PEOE6	6	Molecular charge descriptor calculated using Gasteiger's Partial equalization of orbital electronegativity method

MK14	PEOE3	12	Molecular charge descriptor calculated using Gasteiger's Partial equalization of orbital electronegativity method
	Sum of topological distances between N..N	9	Sum of topological distances between all nitrogen atoms in the molecule
	PEOE5	8	Molecular charge descriptor calculated using Gasteiger's Partial equalization of orbital electronegativity method
	aaN_Key	8	Presence of the aaN (electrotopological state index nomenclature) moiety in the molecule
	aasC_Cnt	7	Number of the aasC (electrotopological state index nomenclature) moieties in the molecule
UROK	PEOE3	12	Molecular charge descriptor calculated using Gasteiger's Partial equalization of orbital electronegativity method
	HBD	11	Number of hydrogen bond donors in the molecule
	Donor groups	7	Number of donor groups in the molecule
	ALOGP2	6	Water-Octanol partition descriptor
	sOH_Key	6	Presence of the sOH (electrotopological state index nomenclature) moiety in the molecule
	aaCH_Cnt	6	Number of the aaCH (electrotopological state index nomenclature) moieties in the molecule
	H-N	6	Number of H-N moieties in the molecule
TRY1	HBD	10	Number of hydrogen bond donors in the molecule
	H-N	10	Number of H-N moieties in the molecule
	ssCH2_Cnt	8	Number of the ssCH2 (electrotopological state index nomenclature) moieties in the molecule
	ALOGP3	6	Water-Octanol partition descriptor

Table S3. A complete listing of all the descriptors appearing in all EOA equations derived for all datasets. For each descriptor, the total number of occurrences is provided.

Dataset	Descriptor	#occurences
ACES	MR1	9
	Balaban-type index from Z weighted distance matrix - Barysz matrix	8
	ssCH2_Avg	6
	Ring bridge count	6
	Sum of topological distances between O..O	5
	sssN_Key	5
	Ring Count 5	5
	PEOE13	5
	Mean topological charge index of order 2	5
	aaCH_Avg	5
	RingCount	4
	dssC_Cnt	4
	dO_Key	4
	ssCH2_Cnt	3
	PEOE9	3
	PEOE12	3
	PEOE11	3
	Num aliphatic rings	3
	MW	3
	Mean topological charge index of order 4	3
	ChiralCenterCount	3
	Average valence connectivity index chi-0	3
	aasC_Avg	3
	RB	2
	Global topological charge	2
	aasC_Cnt	2
	Tertiary amines or amides	1
	Sum of topological distances between N..S	1
	ssO_Key	1
	sCH3_Cnt	1

	sCH3_Avg	1
	PEOE14	1
	Mean topological charge index of order 9	1
	Mean topological charge index of order 8	1
	Maximal electrotopological negative variation	1
	ALOGP6	1
	aaN_Key	1
HIVPR	Neutral acceptor groups	7
	sOm_Key	7
	sssN_Key	7
	aaCH_Avg	6
	aaCH_Cnt	6
	dssC_Cnt	6
	PEOE6	6
	aasC_Avg	5
	dO_Cnt	5
	ssCH2_Cnt	5
	aasC_Cnt	4
	Maximal electrotopological positive variation	4
	Ring Count 5	4
	ChiralCenterCount	3
	dO_Avg	3
	Mean topological charge index of order 10	3
	Sum of topological distances between N..N	3
	aasN_Key	2
	dsCH_Key	2
	Maximal electrotopological negative variation	2
	MW	2
	PEOE3	2
	PEOE9	2
	PSA	2
	sssCH_Key	2
	Total charge	1

	aaN_Key	1
	ALOGP	1
	ALOGP6	1
	ALOGP7	1
	Average eccentricity	1
	dssC_Sum	1
	Global topological charge	1
	HBA	1
	HBD	1
	Mean topological charge index of order 7	1
	Mean topological charge index of order 8	1
	Num aliphatic rings	1
	PEOE10	1
	RingCount	1
	sCH3_Cnt	1
	Sum of topological distances between N..S	1
	Sum of topological distances between O..O	1
MK14	PEOE3	12
	Sum of topological distances between N..N	9
	PEOE5	8
	aaN_Key	8
	aasC_Cnt	7
	ChiralCenterCount	6
	RingCount	5
	PSA	5
	HBD	5
	Ring bridge count	5
	Neutral acceptor groups	4
	dO_Cnt	4
	PEOE6	3
	path/walk 3 - Randic shape index	3
	ssNH_Key	3
	Mean topological charge index of order 3	3

	RB	2
	PEOE11	2
	dO_Avg	2
	aaCH_Cnt	2
	PEOE1	2
	Sum of topological distances between O..S	1
	ssO_Key	1
	ssCH2_Cnt	1
	Ring Count 5	1
	PEOE7	1
	PEOE12	1
	Num aliphatic rings	1
	MW	1
	ALOGP6	1
	ALOGP5	1
	Sum of topological distances between N..S	1
	sssN_Key	1
	PEOE14	1
	PEOE13	1
	Mean topological charge index of order 4	1
	Maximal electrotopological negative variation	1
	Global topological charge	1
	Amide hydrogens	1
	sCH3_Cnt	1
	ALOGP3	1
UROK	PEOE3	12
	HBD	11
	Donor groups	7
	ALOGP2	6
	sOH_Key	6
	aaCH_Cnt	6
	H-N	6
	Mean topological charge index of order 4	4

	ALOGP7	4
	MR1	4
	ALOGP5	3
	PEOE14	3
	ALOGP4	3
	Sum of topological distances between N..O	3
	ssCH2_Cnt	3
	ssNH_Key	3
	MW	2
	Mean topological charge index of order 8	2
	PEOE13	2
	aaCH_Avg	2
	Maximal electrotopological negative variation	2
	Sum of topological distances between N..N	2
	Mean topological charge index of order 10	2
	sNH2_Key	2
	PEOE10	2
	Amide hydrogens	2
	aaN_Key	2
	Average connectivity index chi-1	1
	dssC_Sum	1
	Mean topological charge index of order 6	1
	PEOE7	1
	aasC_Cnt	1
	RB	1
	Balaban-type index from Z weighted distance matrix - Barysz matrix	1
	Mean topological charge index of order 5	1
	PEOE6	1
	ALOGP6	1
	dO_Cnt	1
	Ring bridge count	1
TRY1	HBD	10
	H-N	10

	ssCH2_Cnt	8
	ALOGP3	6
	ALOGP2	5
	MW	5
	aaCH_Cnt	5
	PEOE11	4
	PEOE13	4
	Mean topological charge index of order 4	4
	PEOE3	3
	PEOE14	3
	ALOGP7	3
	MR1	3
	sNH2_Key	3
	Ring Count 5	3
	ALOGP6	3
	PEOE12	2
	PEOE9	2
	Global topological charge	2
	sOH_Key	2
	aaCH_Avg	2
	dssC_Sum	2
	Ring bridge count	2
	sCH3_Avg	2
	Maximal electrotopological negative variation	2
	Sum of topological distances between N..O	1
	Total structure connectivity	1
	Mean topological charge index of order 5	1
	ssNH_Cnt	1
	PEOE10	1
	Balaban-type index from Z weighted distance matrix - Barysz matrix	1
	RB	1
	dO_Avg	1
	sssCH_Sum	1

	ChiralCenterCount	1
	Mean topological charge index of order 7	1
	PEOE7	1
	ALOGP5	1
	ALOGP4	1
	Sum of topological distances between O..S	1
	RingCount	1
	dssC_Cnt	1
	sssN_Key	1
	AlogP	1
	Mean topological charge index of order 6	1

Table S4. EOA and docking results for all test sets expressed in terms of AUC and $EF_{1\%}$ values. The docking results are based on the DUD-E associated crystal structures (1e66, 1xl2, 2qd9, 1sqt, and 2ayw for ACES, HIVPR, MK14, UROK, and TRY1, respectively).

Set	Method	AUC					$EF_{1\%}$				
		**ACES**	**HIVPR**	**MK14**	**UROK**	**TRY1**	**ACES**	**HIVPR**	**MK14**	**UROK**	**TRY1**
1	EOA-7	0.862	0.775	0.905	0.997	0.979	36.449	3.965	40.132	77.551	73.810
	EOA-10	0.886	0.946	0.947	0.997	0.996	26.168	20.705	39.474	81.633	80.159
	EOA-13	0.899	0.977	0.927	0.996	0.986	58.879	59.471	25.658	81.633	58.730
	AD Vina	0.782	0.703	0.727	0.771	0.809	14.019	0.881	7.237	6.122	6.349
	GOLD	0.753	0.665	0.696	0.831	0.808	34.579	9.692	9.211	30.612	11.905
	Glide	0.696	0.607	0.561	0.850	0.772	13.084	4.405	11.184	48.980	11.905
2	EOA-7	0.808	0.813	0.902	0.986	0.958	8.411	14.097	20.395	69.388	75.397
	EOA-10	0.885	0.955	0.914	0.978	0.982	43.925	21.586	41.447	81.633	79.365
	EOA-13	0.921	0.927	0.925	0.987	0.966	9.346	19.383	37.500	73.469	71.429
	AD Vina	0.781	0.694	0.753	0.784	0.805	14.019	2.203	3.947	6.122	4.762
	GOLD	0.738	0.628	0.708	0.830	0.827	31.776	9.251	6.579	32.653	21.429
	Glide	0.651	0.584	0.537	0.817	0.780	15.888	3.084	11.184	40.816	19.841
3	EOA-7	0.896	0.918	0.894	0.955	0.982	42.991	9.692	25.000	79.592	76.984
	EOA-10	0.860	0.946	0.891	0.956	0.980	7.477	34.802	23.684	75.510	79.365
	EOA-13	0.895	0.882	0.918	0.959	0.981	21.495	31.718	27.632	89.796	73.016
	AD Vina	0.795	0.709	0.717	0.803	0.795	15.888	0.441	4.605	8.163	5.556
	GOLD	0.730	0.666	0.696	0.881	0.831	26.168	11.013	7.895	34.694	20.635
	Glide	0.670	0.615	0.573	0.871	0.788	17.757	5.286	15.789	48.980	19.841
4	EOA-7	0.859	0.915	0.892	0.980	0.983	14.019	16.300	21.711	61.224	80.159
	EOA-10	0.919	0.922	0.934	0.986	0.982	58.879	13.656	23.026	67.347	76.190
	EOA-13	0.945	0.978	0.934	0.983	0.983	13.084	51.542	36.184	79.592	79.365
	AD Vina	0.810	0.718	0.747	0.798	0.798	12.150	2.203	7.895	12.245	7.143
	GOLD	0.742	0.642	0.681	0.797	0.802	28.037	10.132	7.237	24.490	19.048
	Glide	0.633	0.611	0.548	0.866	0.791	14.953	5.286	12.500	40.816	26.984

Table S5. EOA and docking results for all test sets expressed in terms of AUC and $EF_{1\%}$ values. The docking results are based on the alternative crystal structures (1acj, 2pwc, 3o8t, 4fue, and 3rxl for ACES, HIVPR, MK14, UROK, and TRY1, respectively).

**Set**	**Method**	**AUC**					**$EF_{1\%}$**				
		**ACES**	**HIVPR**	**MK14**	**UROK**	**TRY1**	**ACES**	**HIVPR**	**MK14**	**UROK**	**TRY1**
1	EOA-7	0.862	0.775	0.905	0.997	0.979	36.449	3.965	40.132	77.551	73.810
	EOA-10	0.886	0.946	0.947	0.997	0.996	26.168	20.705	39.474	81.633	80.159
	EOA-13	0.899	0.977	0.927	0.996	0.986	58.879	59.471	25.658	81.633	58.730
	AD Vina	0.763	0.754	0.712	0.739	0.805	6.542	7.048	9.211	4.082	6.349
	GOLD	0.722	0.734	0.607	0.817	0.872	25.234	14.097	6.579	32.653	23.016
	Glide	0.736	0.682	0.746	0.738	0.813	13.208	12.335	12.500	38.776	37.600
2	EOA-7	0.808	0.813	0.902	0.986	0.958	8.411	14.097	20.395	69.388	75.397
	EOA-10	0.885	0.955	0.914	0.978	0.982	43.925	21.586	41.447	81.633	79.365
	EOA-13	0.921	0.927	0.925	0.987	0.966	9.346	19.383	37.500	73.469	71.429
	AD Vina	0.763	0.762	0.734	0.759	0.784	7.477	3.965	7.895	0.000	6.349
	GOLD	0.705	0.696	0.626	0.743	0.843	21.495	12.775	7.237	24.490	23.016
	Glide	0.675	0.606	0.733	0.773	0.790	8.411	9.292	11.184	32.653	42.400
3	EOA-7	0.896	0.918	0.894	0.955	0.982	42.991	9.692	25.000	79.592	76.984
	EOA-10	0.860	0.946	0.891	0.956	0.980	7.477	34.802	23.684	75.510	79.365
	EOA-13	0.895	0.882	0.918	0.959	0.981	21.495	31.718	27.632	89.796	73.016
	AD Vina	0.758	0.776	0.747	0.750	0.790	5.607	8.370	9.868	6.122	3.175
	GOLD	0.693	0.727	0.608	0.831	0.848	20.561	17.621	8.553	34.694	29.365
	Glide	0.698	0.654	0.742	0.787	0.817	10.280	9.778	13.816	38.776	42.857
4	EOA-7	0.859	0.915	0.892	0.980	0.983	14.019	16.300	21.711	61.224	80.159
	EOA-10	0.919	0.922	0.934	0.986	0.982	58.879	13.656	23.026	67.347	76.190
	EOA-13	0.945	0.978	0.934	0.983	0.983	13.084	51.542	36.184	79.592	79.365
	AD Vina	0.791	0.785	0.741	0.764	0.805	4.673	5.727	4.605	8.163	3.968
	GOLD	0.714	0.731	0.615	0.791	0.859	16.822	14.097	7.237	28.571	26.984
	Glide	0.693	0.630	0.742	0.761	0.817	10.280	12.335	10.526	40.816	47.200

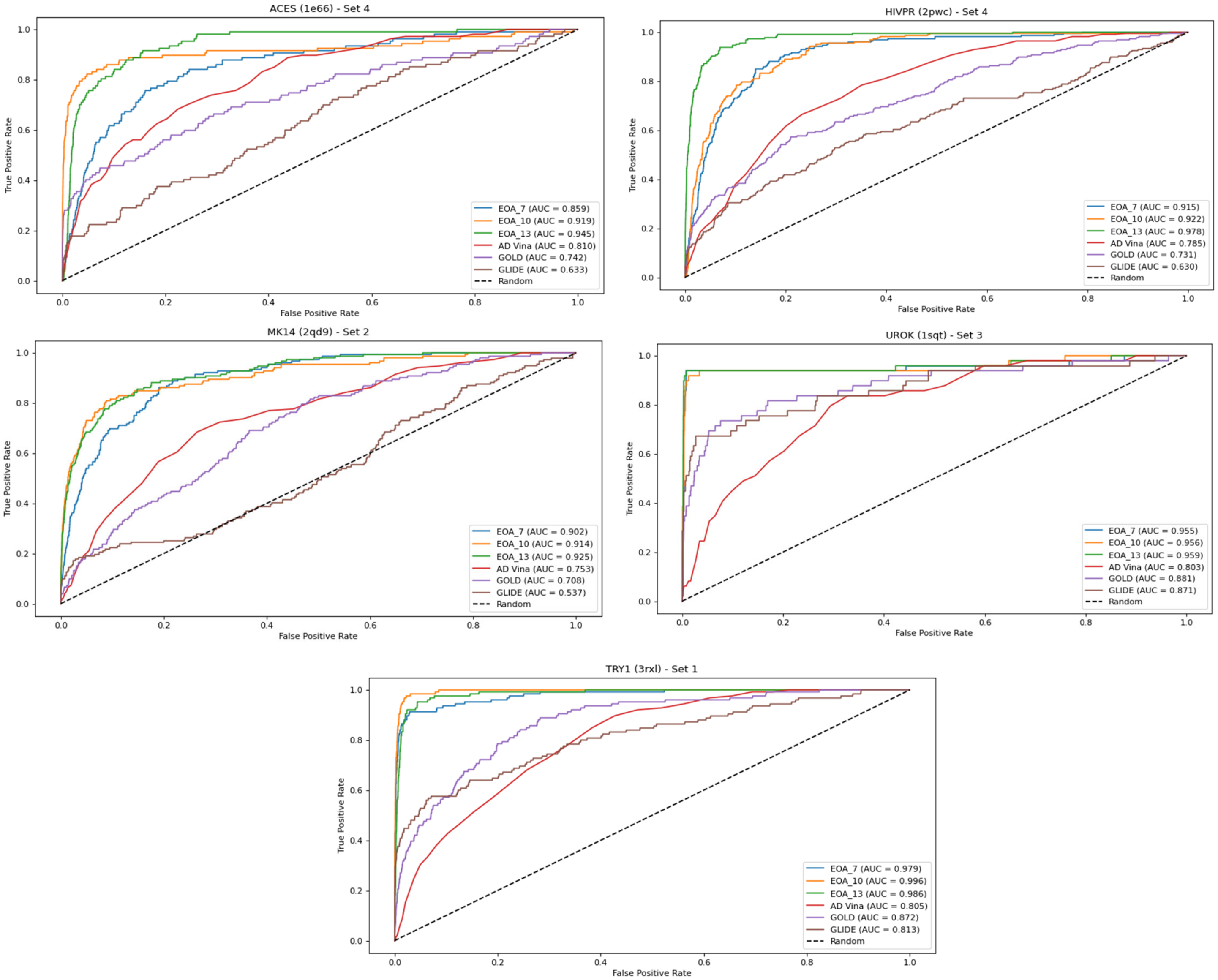

Figure S1. ROC curves for the best-performing docking method (across the two structures and the four subsets) compared with EOA and the other docking methods (see text for more details). In all cases, the EOA performed better than all docking methods.

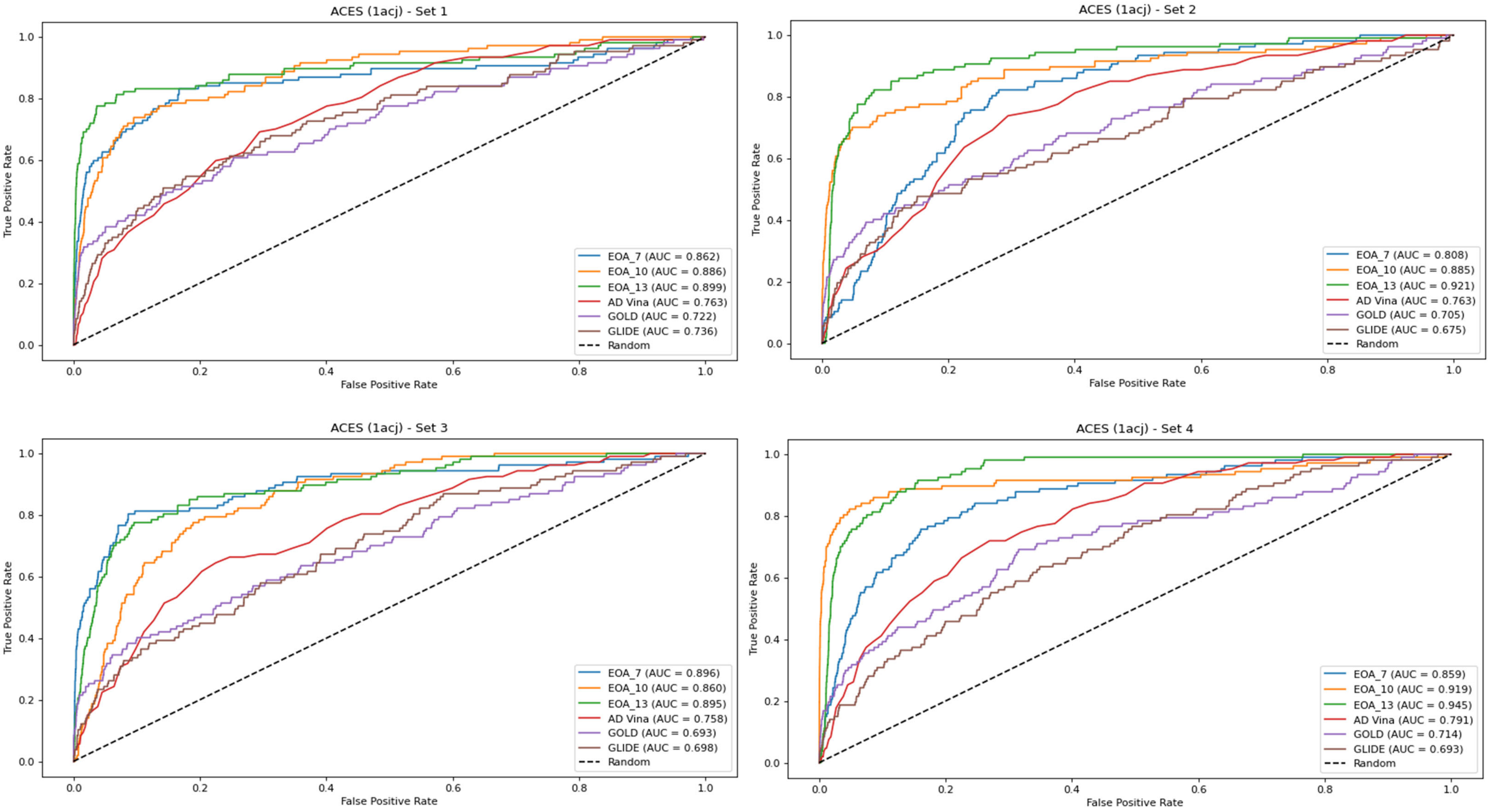

Figure S2. ROC curves for the four ACES (1acj) subsets.

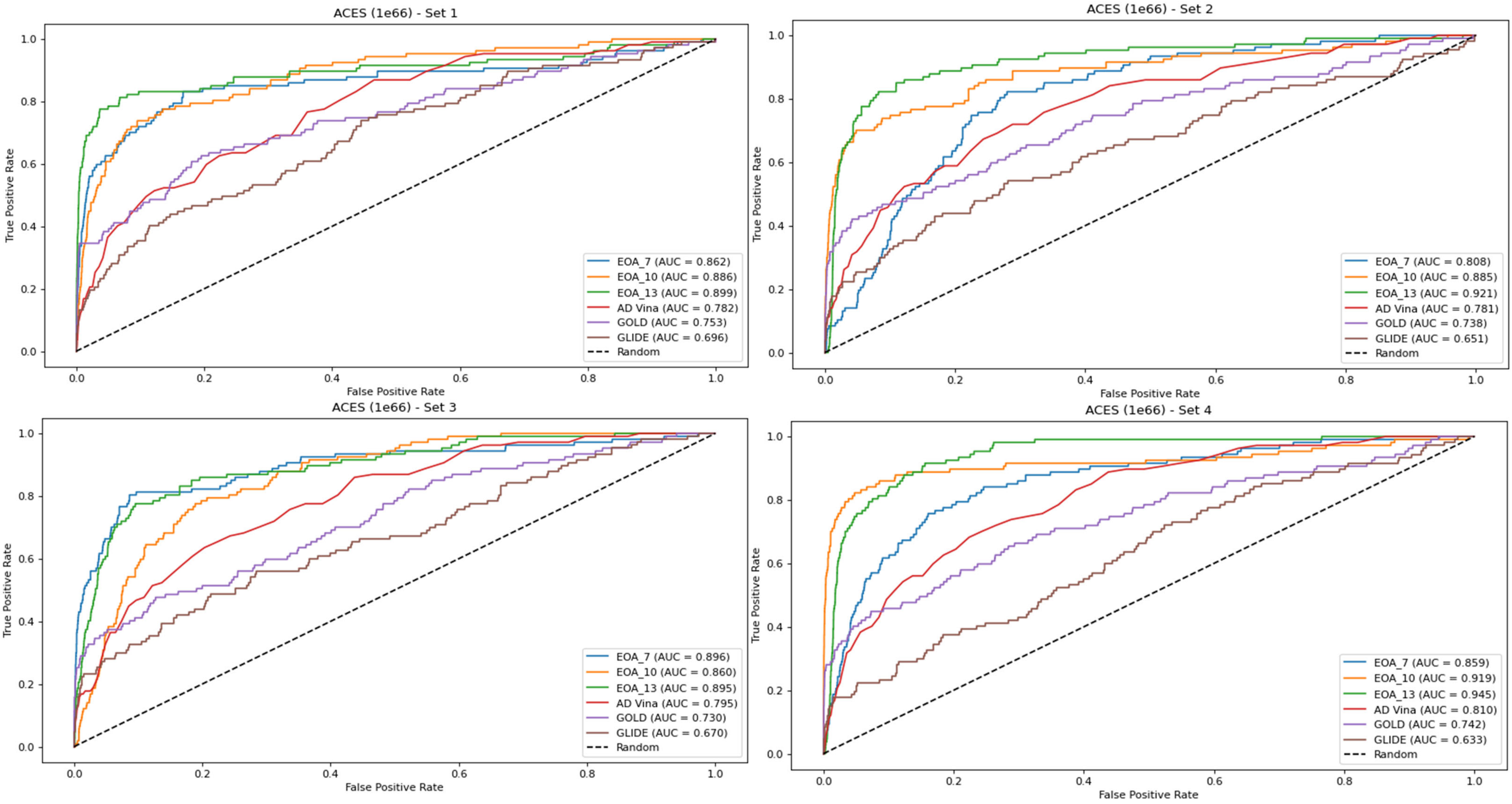

Figure S3. ROC curves for the four ACES (1e66) subsets.

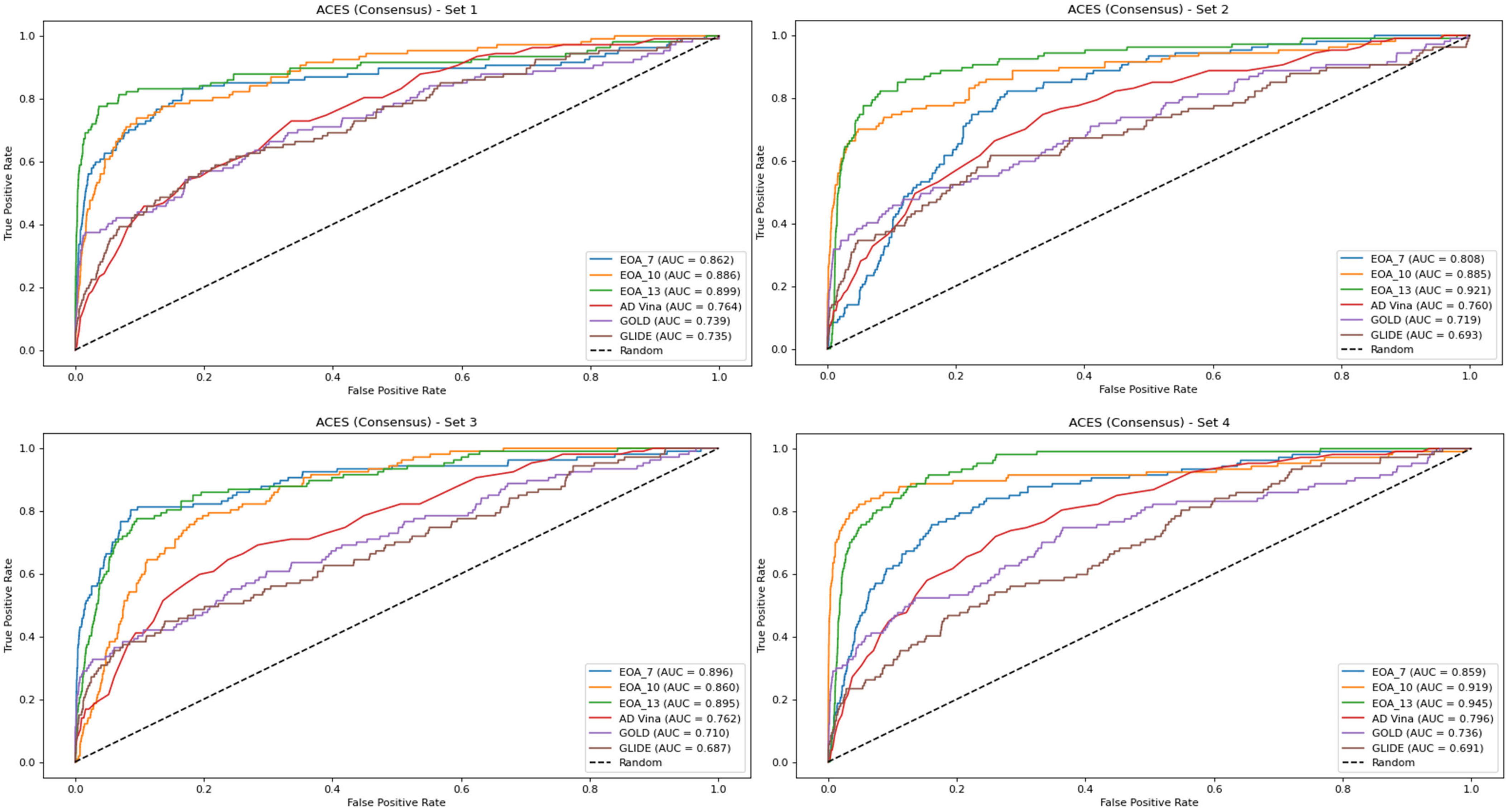

Figure S4. ROC curves for the four ACES (Consensus) subsets.

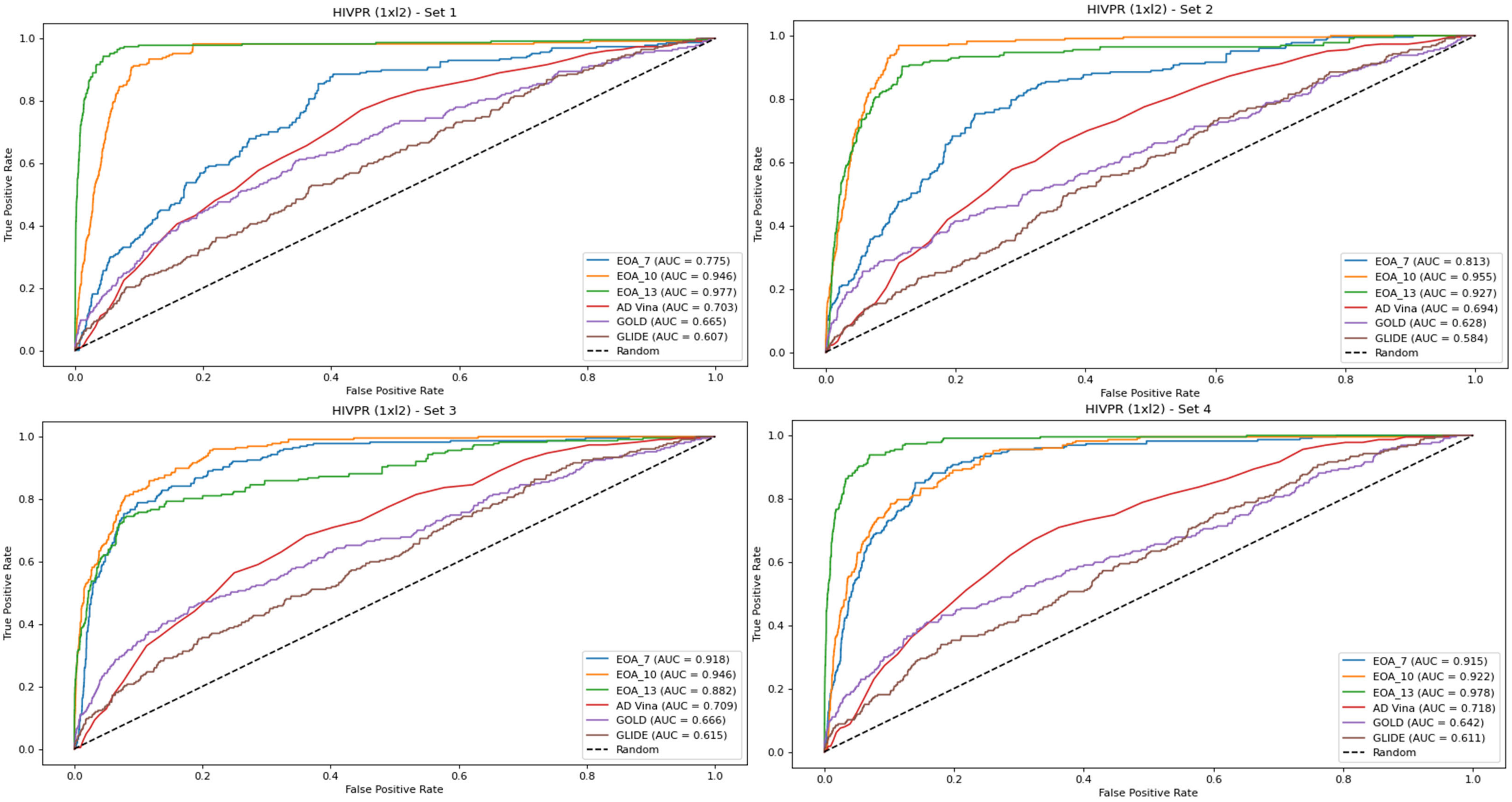

Figure S5. ROC curves for the four HIVPR (1xl2) subsets.

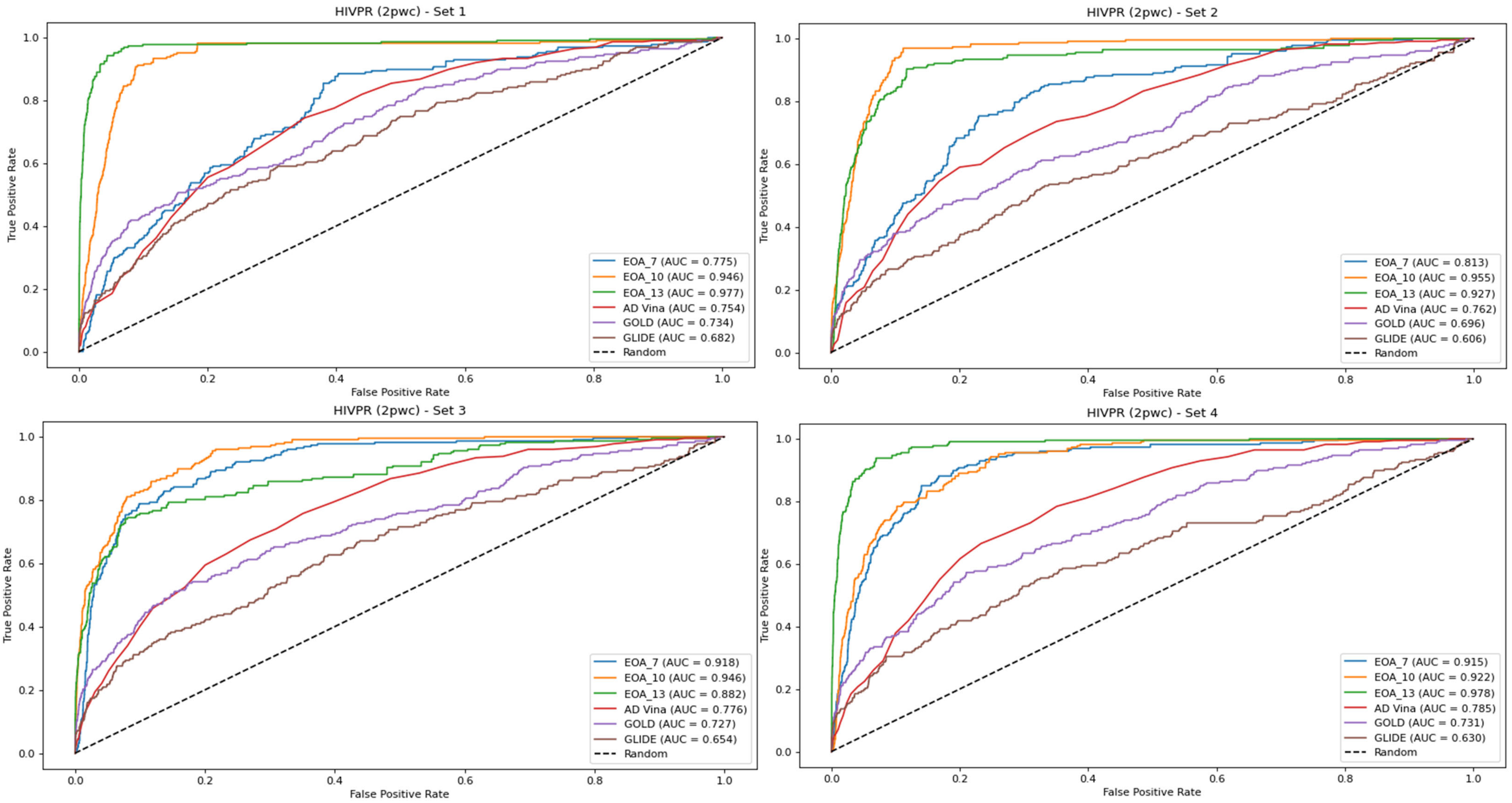

Figure S6. ROC curves for the four HIVPR (2pwc) subsets.

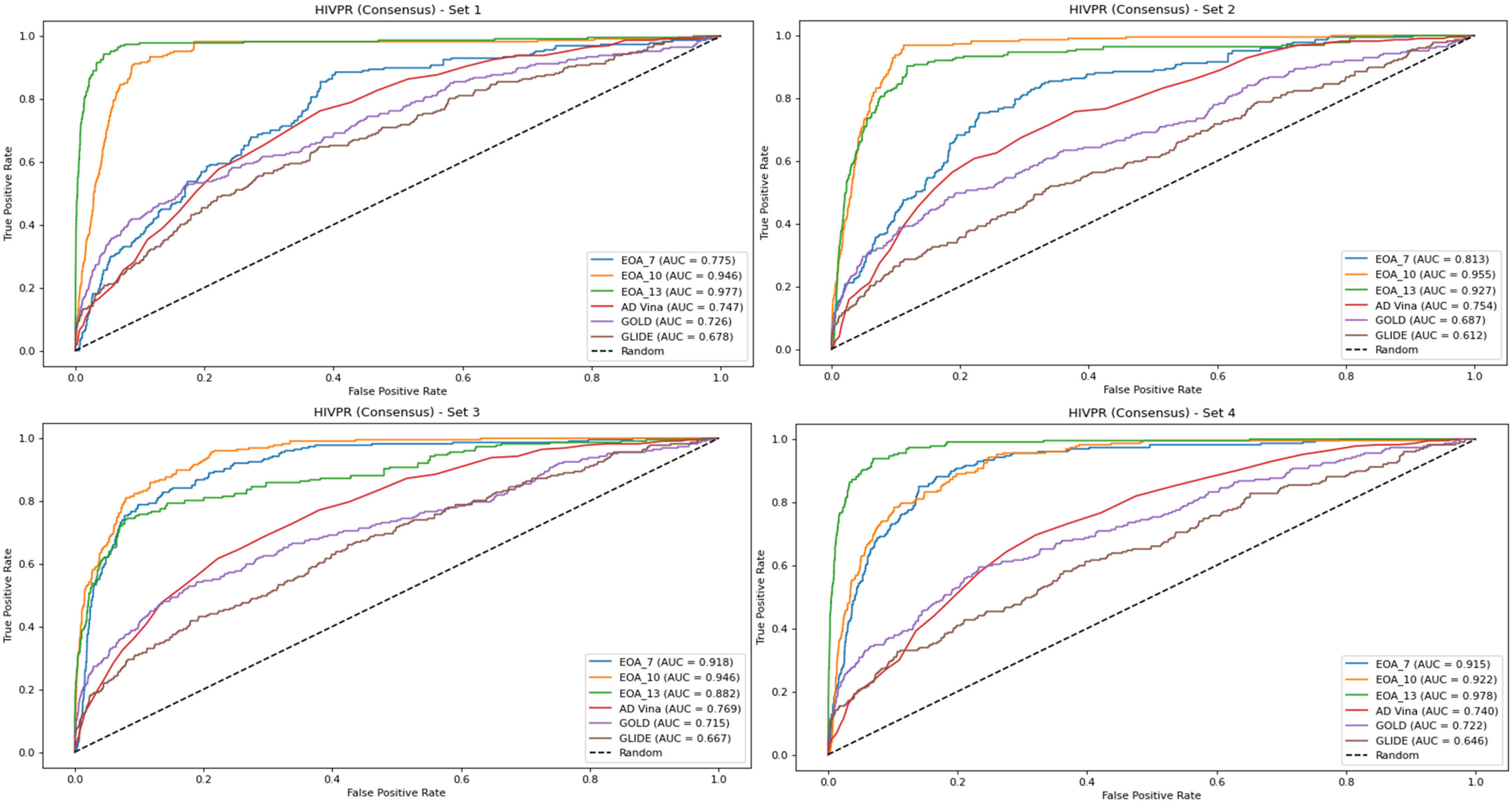

Figure S7. ROC curves for the four HIVPR (Consensus) subsets.

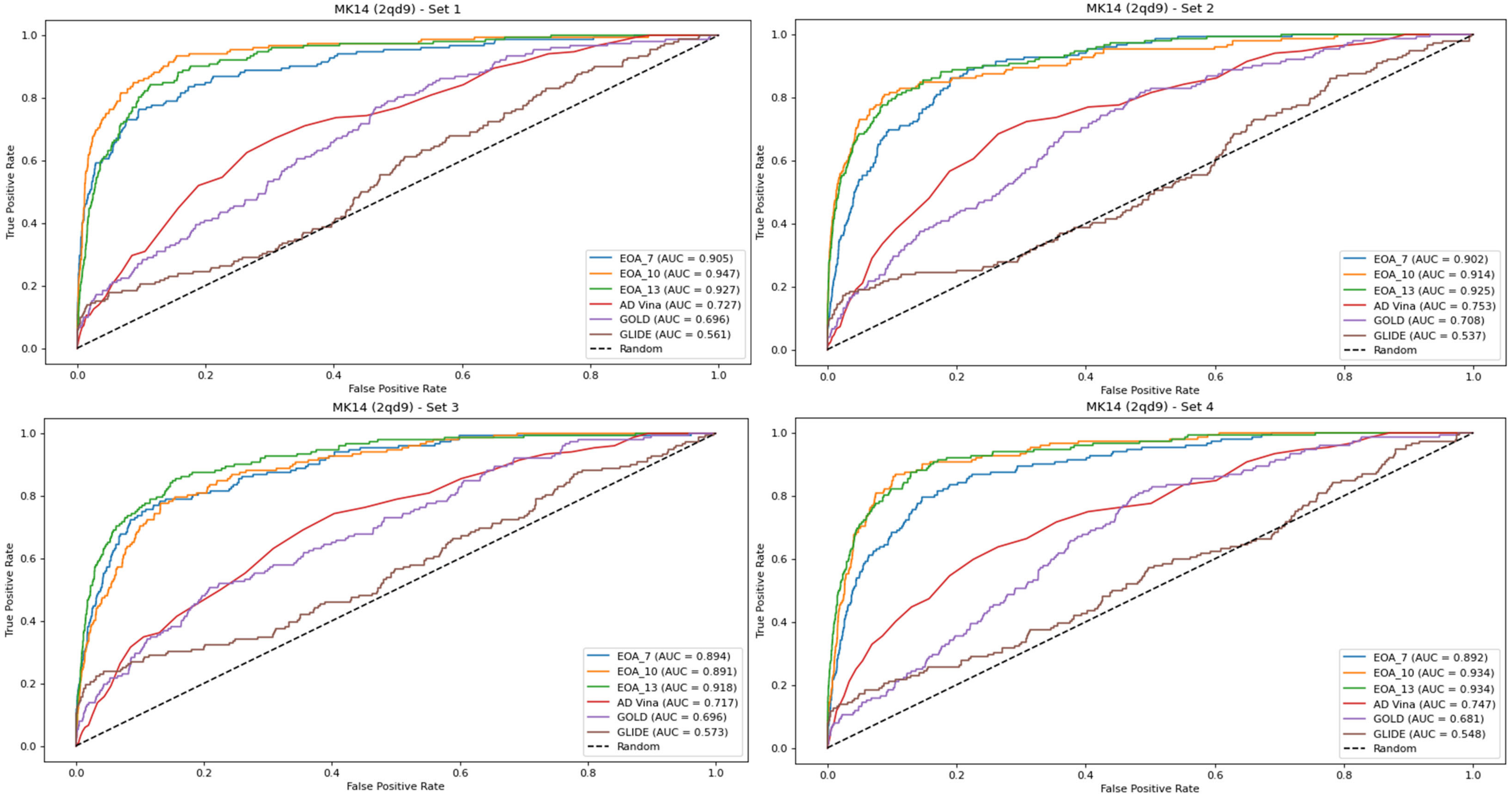

Figure S8. ROC curves for the four MK14 (2qd9) subsets.

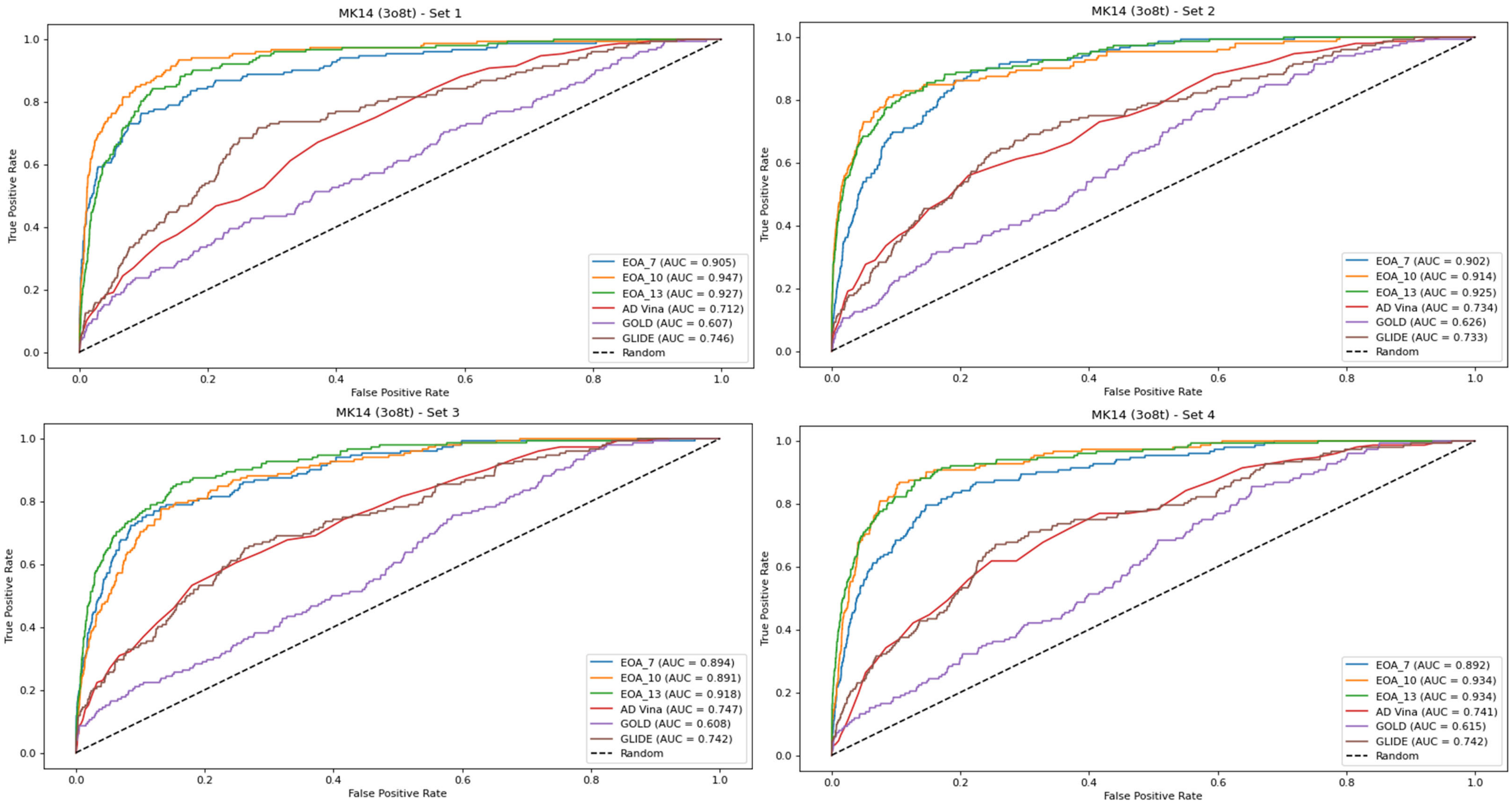

Figure S9. ROC curves for the four MK14 (3ot8) subsets.

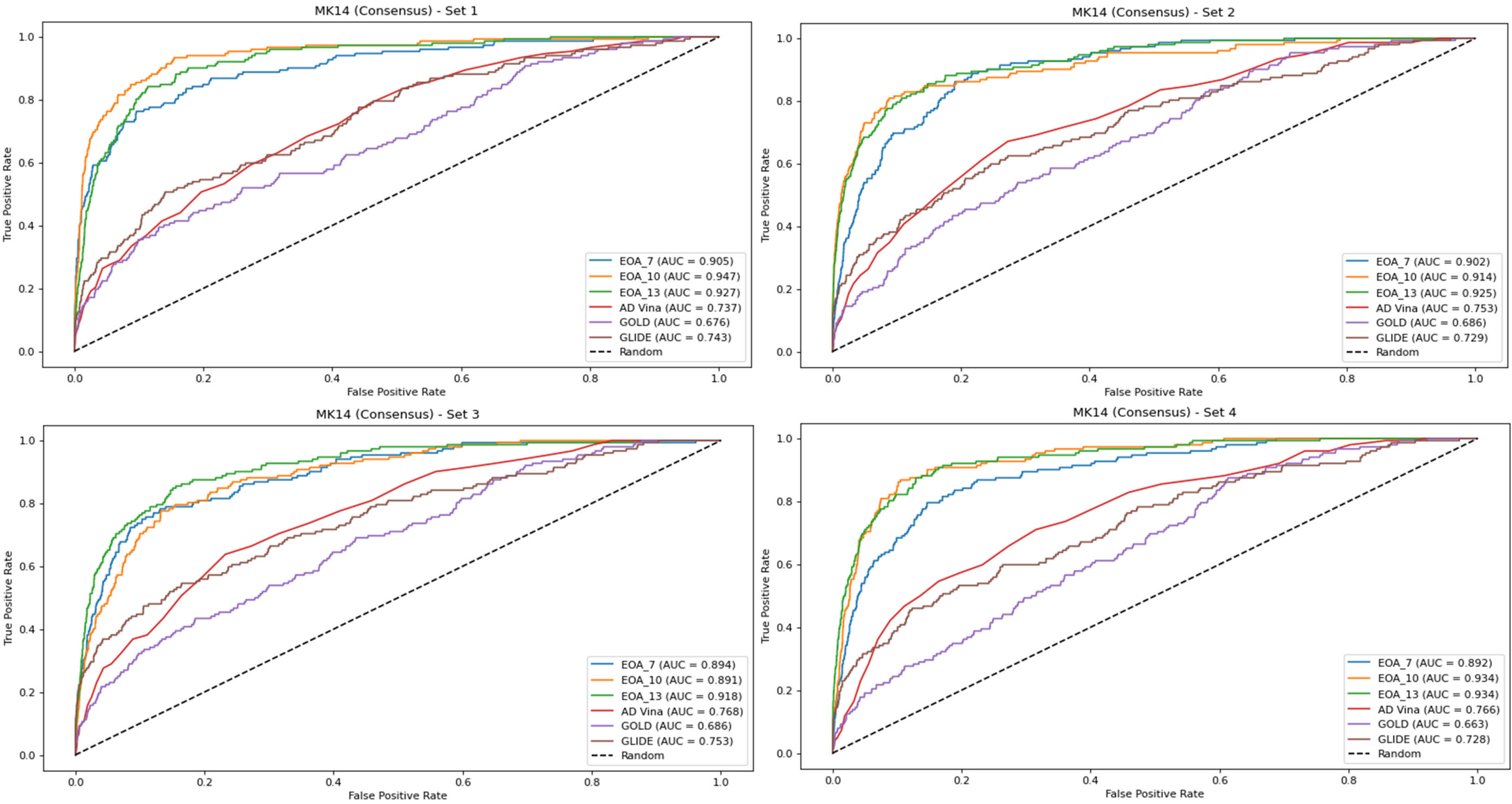

Figure S10. ROC curves for the four MK14 (Consensus) subsets.

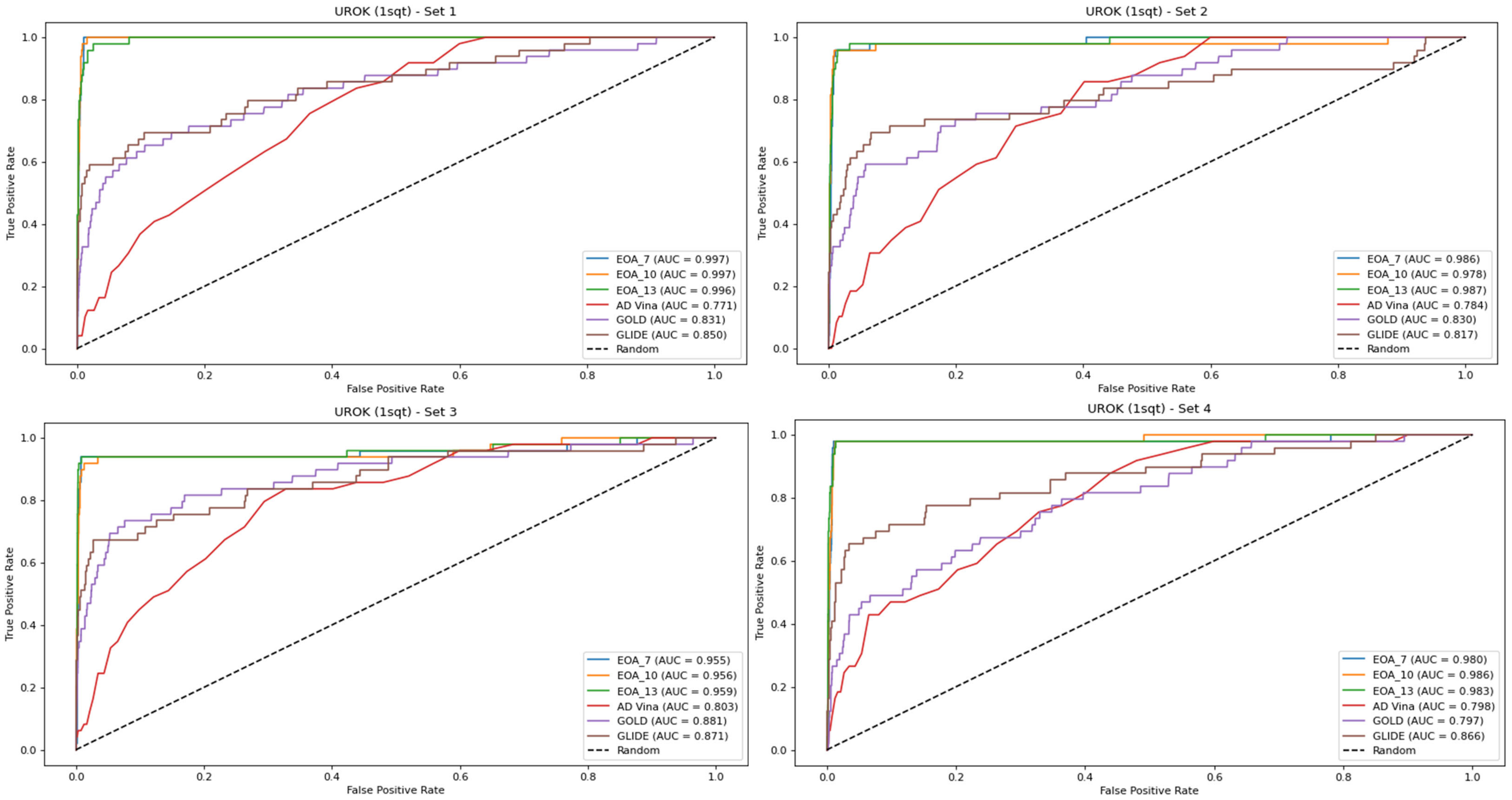

Figure S11. ROC curves for the four UROK (1sqt) subsets.

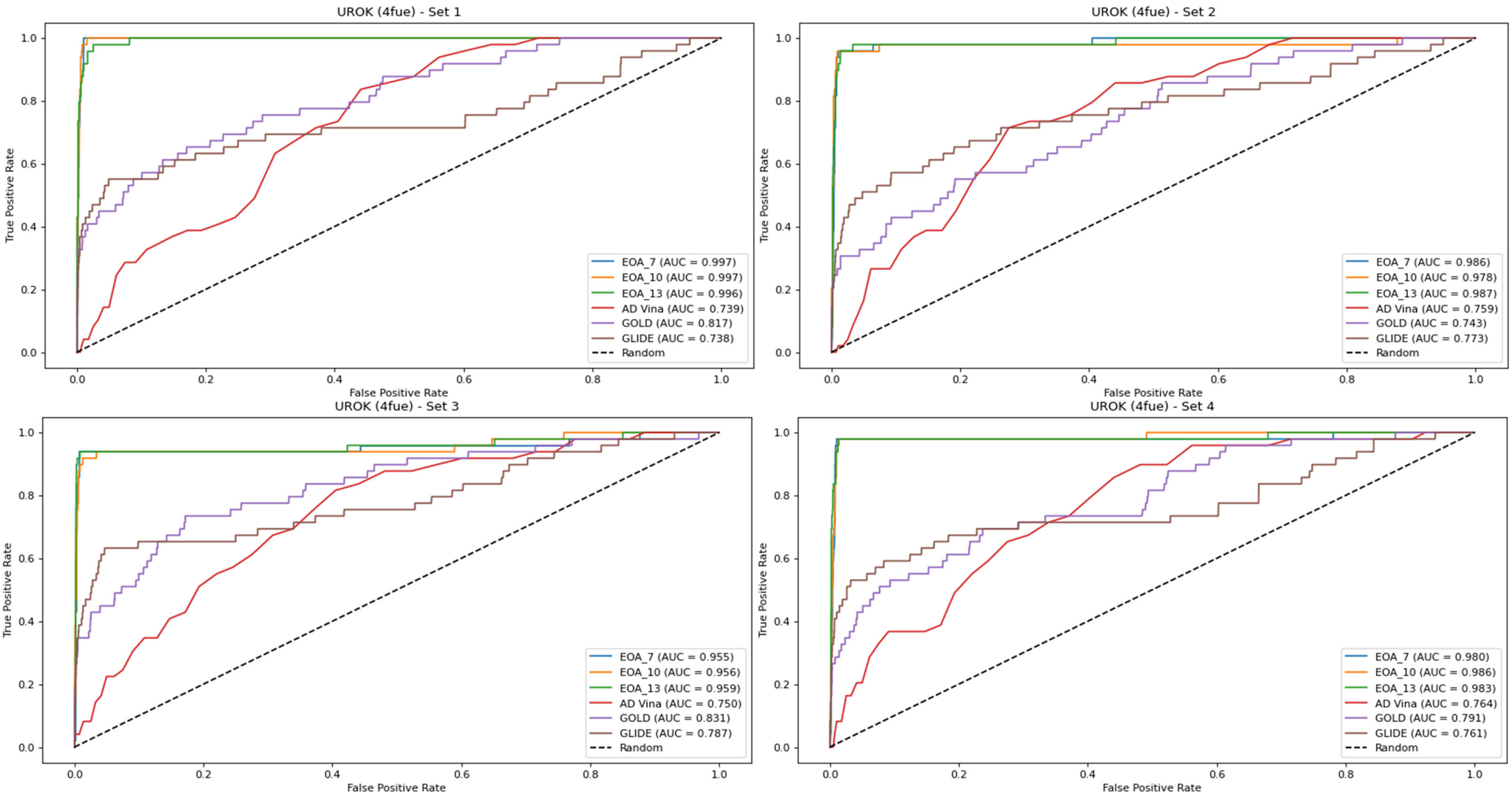

Figure S12. ROC curves for the four UROK (4fue) subsets.

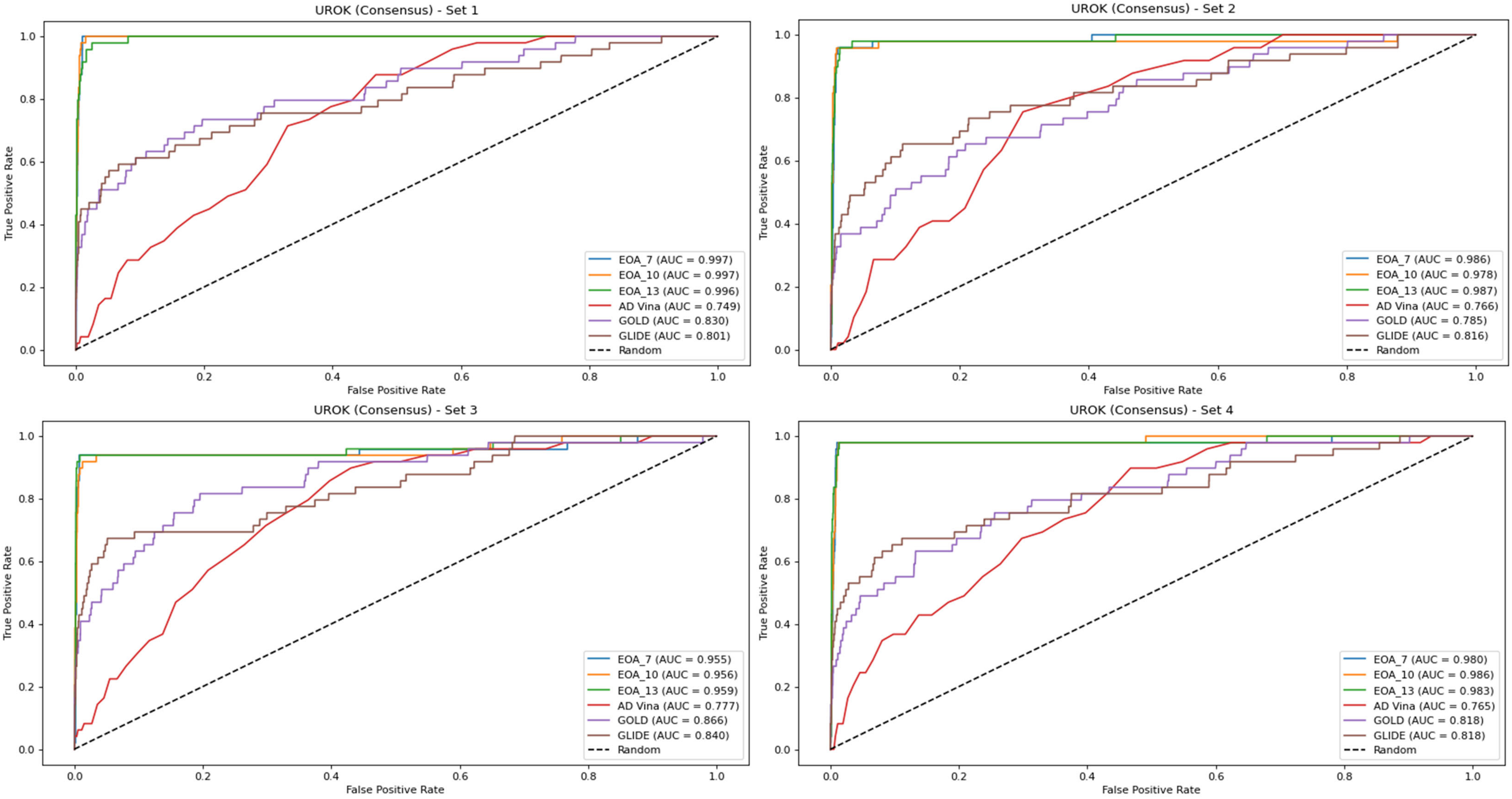

Figure S13. ROC curves for the four UROK (Consensus) subsets.

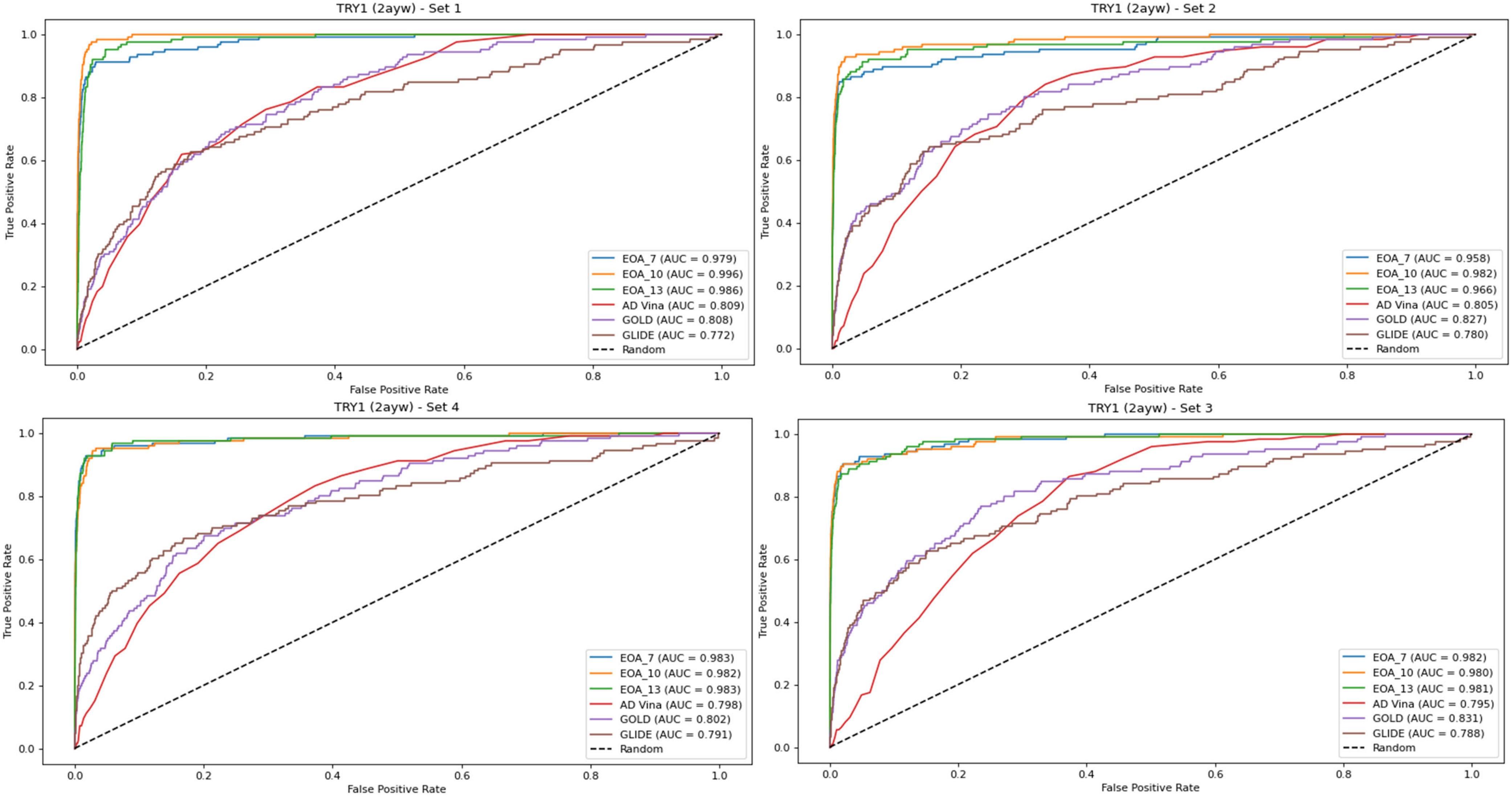

Figure S14. ROC curves for the four TRY1 (2ayw) subsets.

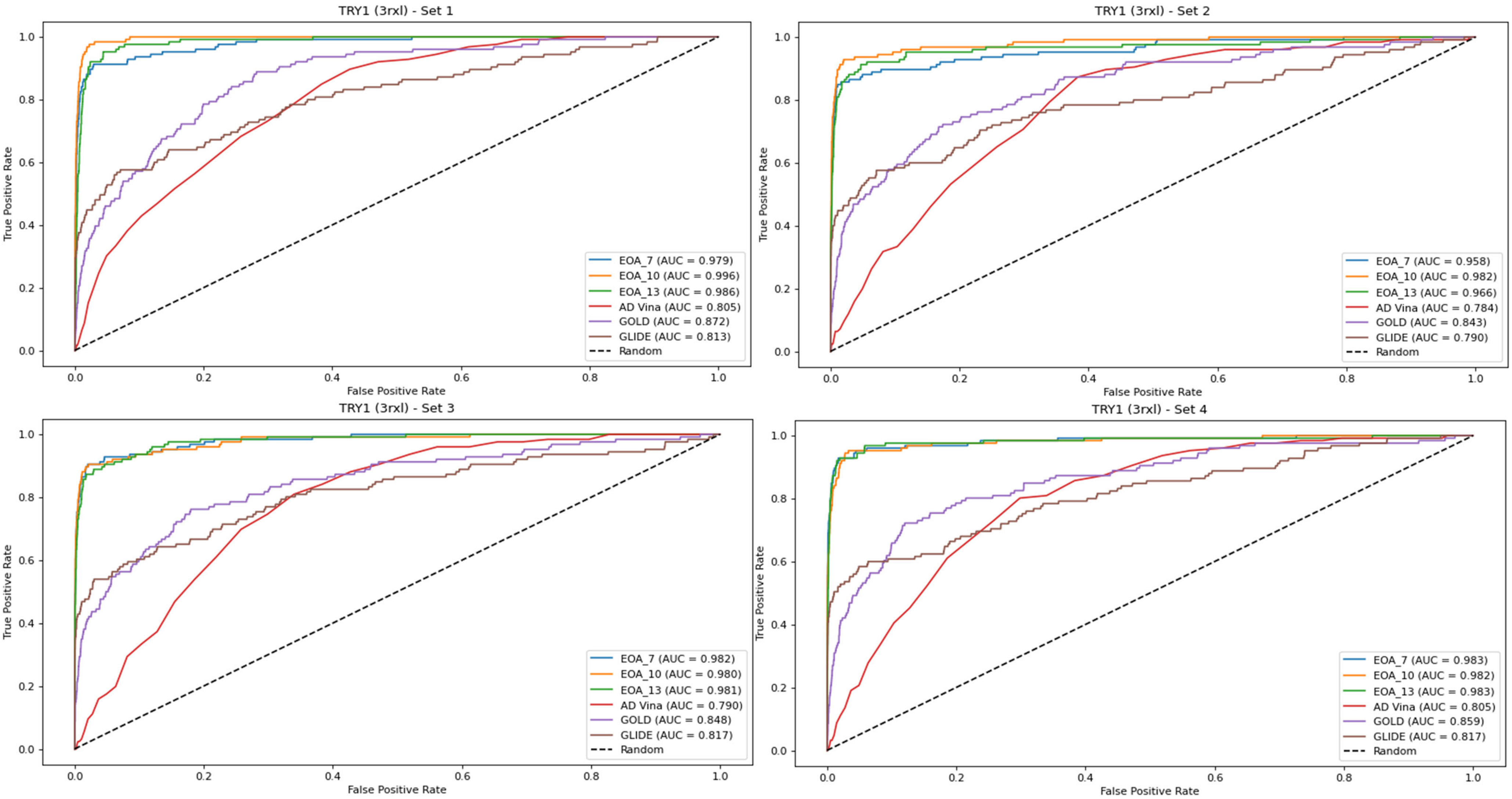

Figure S15. ROC curves for the four TRY1 (3rxl) subsets.

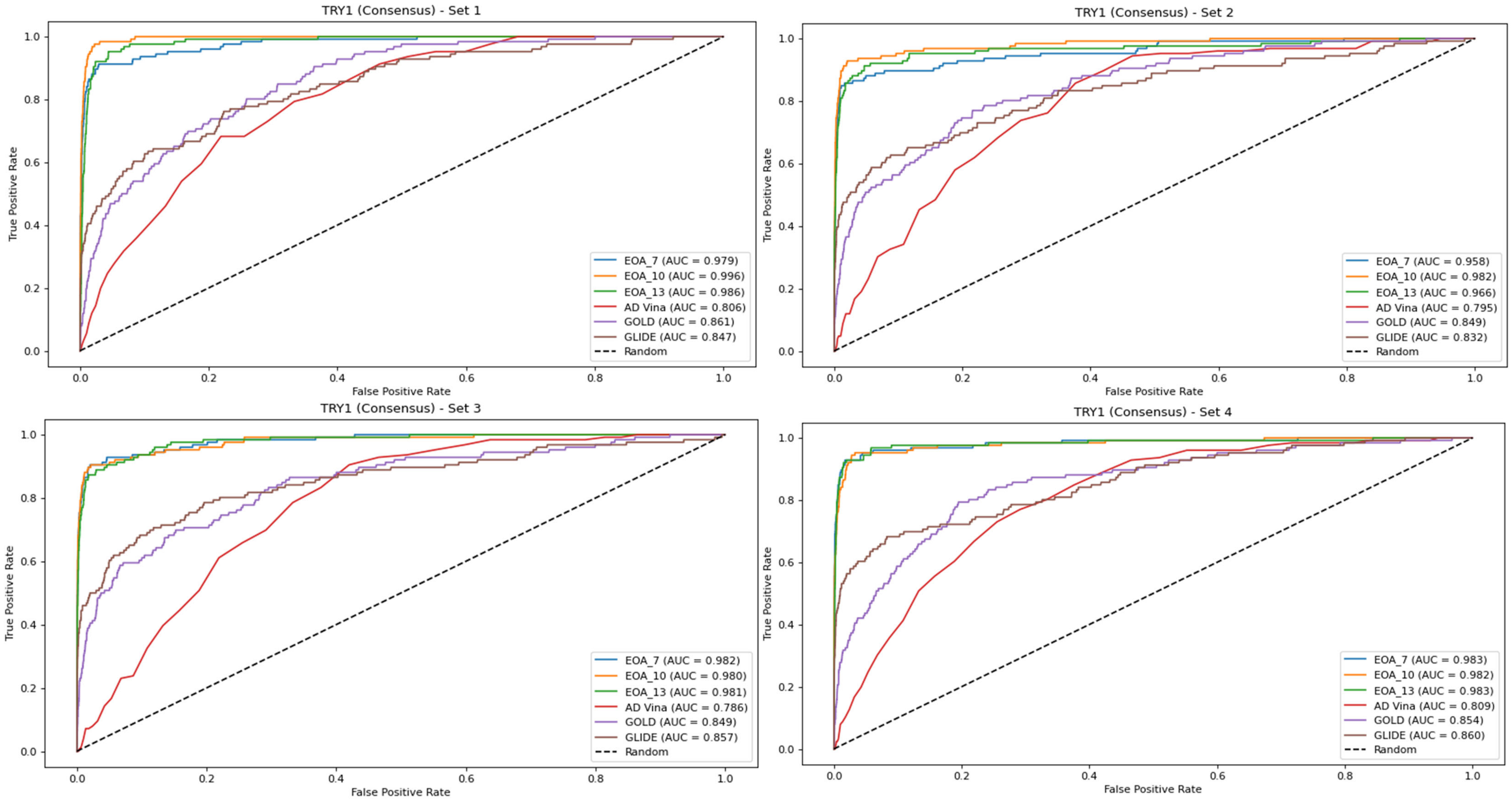

Figure S16. ROC curves for the four TRY1 (Consensus) subsets.
